# Editorial: Women in chemistry 2022

**DOI:** 10.3389/fchem.2023.1230005

**Published:** 2023-10-06

**Authors:** Irene S. Fahim, Vânia André, Jyotirmayee Mohanty, Rosalia Maria Cigala, Srabanti Ghosh, Luísa M. D. R. S. Martins, Weiwei Han, Anna Maria Papini, Joana Costa, Maela Manzoli, Ottavia Giuffrè, Reshma Rani, Sarish Rehman, Debbie C. Crans, Gabriela Oksdath-Mansilla, Federica Sabuzi

**Affiliations:** ^1^ Industrial Engineering Program, Director Smart Engineering Systems Research Center, Nile University, Giza, Egypt; ^2^ Centro de Química Estrutural, Institute of Molecular Sciences, Instituto Superior Técnico, Universidade de Lisboa, Lisbon, Portugal; ^3^ Associação Do Instituto Superior Técnico para a Investigação e Desenvolvimento, Lisbon, Portugal; ^4^ Bhabha Atomic Research Centre, Trombay, India; ^5^ Homi Bhabha National Institute, Training School Complex, Mumbai, India; ^6^ Department of Chemical, Biological, Pharmaceutical and Environmental Sciences, University of Messina, Messina, Italy; ^7^ Energy Materials and Devices Division, CSIR—Central Glass and Ceramic Research Institute, Kolkata, India; ^8^ Centro de Química Estrutural, Institute of Molecular Sciences, Departamento de Engenharia Química, Instituto Superior Técnico, Universidade de Lisboa, Lisbon, Portugal; ^9^ Key Laboratory for Molecular Enzymology and Engineering of Ministry of Education, School of Life Sciences, Jilin University, Changchun, China; ^10^ Interdepartmental Research Unit of Peptide and Protein Chemistry and Biology, Department of Chemistry “Ugo Schiff”, University of Florence, Sesto Fiorentino, Italy; ^11^ Centre for Functional Ecology, Associate Laboratory TERRA, Department of Life Sciences, University of Coimbra, Coimbra, Portugal; ^12^ Department of Drug Science and Technology and NIS Centre, University of Turin, Turin, Italy; ^13^ Drug Discovery Chemistry, Jubilant Biosys, Noida, Uttar Pradesh, India; ^14^ Department of Chemistry, McGill University, Montreal, QC, Canada; ^15^ Department of Chemistry, Colorado State University, Fort Collins, CO, United States; ^16^ Instituto de Investigaciones en Físico-Química de Córdoba (INFIQC-CONICET), Departamento de Química Orgánica, Facultad de Ciencias Químicas, Universidad Nacional de Córdoba, Ciudad Universitaria, Córdoba, Argentina; ^17^ Department of Chemical Science and Technologies University of Rome Tor Vergata, Roma, Italy

**Keywords:** bioplastics, photocatalyst, waste valorization, oxidative stress, anticancer agents, antimicrobial peptide, luminescent material, hydrogel

## Introduction

A wide plethora of research exists on women in STEM education, but research by female chemists in educational and employment settings is scarce, especially in the Middle East and North Africa region. Most current industries use computer-driven machinery, and rising levels of automation and automated control systems that are driven by machines have altered shop floor procedures. The necessary qualifications and skills for chemistry jobs have changed as a result.

Chemistry education in Egypt is advancing. At least 15 STEM high schools operated by the Education Ministry have been established since 2011. In 2017, 43% of Egypt’s university STEM students were women which is higher than the percentage in the UK and all over the globe. The percentage increased to reach almost 47% in 2018/2019 as was reported in the Higher education bulletin from CAPMAS. The percentage of female graduates by field is shown in the 2021 UNESCO scientific report using data from 2018: agriculture 49.4%, engineering 20.9%, health & welfare 56%, natural sciences 64.2%, and ICT 36.8%. According to UNESCO data, in 2018 only 46% of Egypt’s total number of scientific researchers were women. This percentage exceeds the average percentage of female researchers in the Arab World which accounts for around 43% and that of EU which accounts for 34%.

Women must engage in all fields in order for societies to advance and stay up with the times. However, in some fields, such as the male-dominated field of Mechanical and Electrical Engineering, there is very little female representation, so we are striving to raise awareness among youth in society.

This editorial plays a small part in this by documenting women’s work in chemistry with all of its qualitative ramifications, highlighting successful research stories, and focusing on advancing and sustaining the presence in public and private sector institutions to emphasize the significance of women working in this profession and empowering them in the field of Chemistry.

Diverse international representation of researchers have contributed to this Research Topic in the 2022 edition and all the manuscripts were published after a thorough peer review process. The following is a summary of the areas and contributions in each of these manuscripts organized in terms of broad research topics.

### Analytical chemistry

This section includes 2 articles involving some areas of Analytical Chemistry, as bioplastic analysis and food products. Censi et al. reported on bioplastics, defined as materials obtained from biomass sources and recycled food waste. They can be divided into biodegradable, bio-based, or both. This minireview described the different methods for bioplastic qualitative analysis, such as fluorescence, Nuclear Magnetic Resonance (NMR), Fourier-Transform Infrared Spectroscopy (FTIR), UV-VIS spectrophotometry, Differential Scanning Calorimetry (DSC), Thermogravimetric Analysis (TGA) and for bioplastic quantitative analysis, such as Gas Chromatography (GC), High-Performance Liquid Chromatography (HPLC), TGA associated with Mass Spectrometry (TGA-MS). The processes of biopolymer degradation were also examined considering the advantages and disadvantages of each method, and their potential environmental impact.

The determination of trace levels of organic fining agents in wines was reported by Bongiorno et al. Fining agents were commonly added to wines to reduce or remove some undesirable compounds, such as proteins, phenols, and tannins. The residues of fining agents should not be present in the final product, any trace residues must be quantified using detection methods. Among them there are immunochemical assays such as Enzyme-Linked Immunosorbent Assay (ELISA), for the detection of exogenous protein residues; Mass Spectrometry (MS) based methods for the determination of contaminants, antioxidants, polymers, surfactants, and proteins; biosensors for the detection of food allergens as lysozyme, ovalbumin, caseins, lactoglobulin.

### Photocatalysis and photochemistry

Research advances in photocatalysis towards sustainable technology for converting solar energy into fuels, mainly through the solar-driven conversion of water and CO_2_ into fuels and chemicals.Many important photocatalytic reactions, i.e., Water splitting, hydrogen evolution, oxygen reduction, CO_2_ reduction, and organic reaction, have been explored. Mainly the photocatalyst has been considered a significant player, and a wide range of photocatalyst materials, from oxides, perovskites, (oxy) halides, organic semiconductors (graphitic carbon nitrides, conjugated polymers, porous polymers, covalent organic framework, etc.), porous materials, and 2D materials for photocatalytic fuel production. Traditional metal oxides are widely utilized photocatalysts but are limited with poor efficiency and absorb light in the short wavelength range. In recent years, significant progress has been achieved in improving the performance of oxide semiconductors by increasing the exposed active areas. A series of new visible light active photocatalysts such as TiO_2_, BiVO_4_, WO_3_, Fe_2_O_3_, Cu_2_O, spinel oxides, perovskites, etc.,.have been developed through the integration of binary or ternary structured photocatalyst loaded with co-catalysts which lower photoexcited charge carriers recombination and achieve high catalytic efficiency and photo-stability. The photocatalytic performance is essentially dependent on the electronic structure of photocatalysts, and the conversion efficiency could be enhanced via rational catalyst design, such as introducing vacancies and constructing a heterointerface. In spite of significant progress in materials development, the current status of the photocatalysts is limited to practical applications from laboratory-scale to industrial-scale. Moreover, various photoreactor systems have been developed for low-cost solar fuel production, which is promising for realizing the commercialization of such technologies. This Research Topic features one review and one original research paper on this Research Topic.

The first article focuses on the synthesis of Catalyst: The Development Synthesis of Tetrahydrobenzo [b]pyran Scaffolds via a Single-Electron Transfer/Energy Transfer Farzaneh Mohamadpour. The authors report on methylene blue (MB+)-derived photo-excited state functions was employed in an aqueous solution as single-electron transfer (SET) and energy transfer catalysts. The formation of malononitrile, and dimedone, a radical tandem Knoevenagel–Michael cyclocondensation reaction of tetrahydrobenzo [b] pyran scaffolds, were developed. A gram-scale cyclization is possible, implying that the technology may be applied in industries.

The review by Wang and Kowalska is focused on the use Property-governed performance of platinum-modified titania photocatalysts. The preparation of platinum deposited on the surface of titania has been shown to improve photocatalytic performance both under UV and visible light. Interestingly, the property governed activity for Pt/TiO_2_ photocatalysts such as platinum content, platinum properties (size, aggregation/uniform distribution, oxidation state), titania properties (size, crystallinity, specific surface area, polymorphic form, defect density, morphology, surface characteristics) and the contact between platinum and titania have been considered an highlighted.

### Chemical biology

In this study, Lee and Heffern investigated the interface between the biological activity of flavonoids and their interaction with copper ions as potential therapeutic targets. Flavonoids are small polyphenolic molecules that are widely distributed in plant products, are known to have beneficial health effects and exhibit complex behaviour in biological systems. Copper contributes to the proper functioning of biological systems and its deregulation is associated with several metabolic diseases. Spectroscopic techniques were used to study the binding of flavonoids to Cu(II) and their radical scavenging activities. The authors identified important structural groups involved in this interaction related to ring substitution. The biological effects of flavonoids on copper transport were also evaluated and shown to be influenced by their hydrophobicity. In conclusion, relevant aspects of the influence of flavonoids on copper transport have been elucidated, contributing to new dietary recommendations and potential therapeutic development.

Zhang et al. showed that the potential drug molecule ^OH^Py_2_N_2_ is the first example of a low molecular weight, macrocyclic small molecule capable of activating cellular mechanisms of protection against oxidative stress. The results reported clearly show that there are multiple pathways that ^OH^Py_2_N_2_ may impact to promote natural mechanisms within cells that protect against chronic oxidative stress in the eye. The work is a paradigmatic example of the contribution of chemical biology, combining the organic and inorganic design of small molecules with studies aimed at elucidating their potential to impact eye disease. Only such a multidisciplinary approach will, soon, be able to characterise the structural components of the ^OH^Py_2_N_2_ molecule responsible for the activation of these pathways and hopefully lead to the identification of non-surgical therapeutic options whose benefits outweigh the risks of cataract surgery complications that may still occur, especially in patients of advanced age.

### Electrochemistry

This Research Topic features two original research papers on the Electrochemistry section. The first one is entitled “Toward practical Research Topic: Identification and mitigation of the impurity effect in glyme solvents on the reversibility of Mg plating/stripping in Mg batteries,” which addresses the challenges associated with the reversible electrochemical plating and stripping of magnesium in magnesium batteries. The authors focus on ether glyme solutions containing the MgTFSI2 salt, which are commonly used electrolytes for achieving reversible behavior of Mg electrodes. The study systematically identifies impurities in these systems and investigates their effects on the Mg deposition-dissolution processes. This study evaluates various mitigation methods to eliminate impurities and discusses their beneficial effects on improved reactivity. Ultimately, the authors propose a conditioning protocol that can be easily implemented and safer for practical applications of MgTFSI2/glyme electrolytes containing impurities. In this article, the authors emphasize the significance of glymes as solvents and MgTFSI2 as a promising salt for Mg batteries, which makes a significant contribution to the field of Mg batteries by addressing the impurity effect in glyme solvents and providing practical strategies for their mitigation. The systematic identification of impurities, evaluation of mitigation methods, and proposal of a conditioning protocol demonstrate the author’s thorough research and understanding of the subject matter. This study’s findings can potentially facilitate the development of practical and efficient electrolyte systems for Mg batteries.

The second paper submitted to this section is entitled: Electrodeposited Fe on Cu foam as advanced Fenton reagent for catalytic mineralization of methyl orange,” which addresses the significant Research Topic of textile dye pollution in water and proposes an innovative solution using Fe/Cu catalysts in heterogeneous Fenton’s reaction. This study uses environmentally friendly electrodeposition techniques to explore the degradation of methyl orange, a model azo dye, in aqueous solutions. In this work, the authors have discussed the advantages of bimetallic Fenton reagents, specifically Fe/Cu catalysts, regarding their catalytic efficiency and stability. They also explain how immobilizing iron species onto porous templates like metal foam can enhance the catalyst’s activity. The use of metal foam as a substrate is justified based on its durability, ductility, and highly effective surface area. This study offers valuable insights into using Fe/Cu catalysts on copper foam as an effective solution for the degradation of textile dyes in wastewater. The research findings, experimental approach, and the environmentally friendly nature of the proposed method make it a significant contribution to the field of wastewater treatment.

### Green and sustainable chemistry

Gender inequality, the fifth sustainable development goal outlined by the United Nations is considered as a significant hindrance to sustainable development and without fully addressing it, reaching the other goals will be much more difficult. Within this context, women play a pivotal role in contributing to improve economic, social, and environmental Research Topic crucial for achieving a sustainable future. Green chemistry is a continuously evolving frontier, which embraces the development of chemical processes and environment-friendly products alternative to hazardous substances to prevent pollution. The extensive use of crude oil raised the need for more sustainable feedstock for energy and materials, which has to meet the growing demand for goods. The design of sustainable chemical processes reduces waste and demand on diminishing resources. In this frame, waste lignocellulosic biomass represents a sustainable alternative feedstock to fossil resources for fuels and chemicals. Indeed this frame, it is possible to obtain innovative materials starting from wastes, for example, humic and fulvic acids formulate nanohybrids with silica obtained from rice husks can be used for agricultural applications and circular processes involving urban waste valorization (Giordana et al.). Another possibility is to employ added value molecules from renewable resources to optimize the production of chemicals usually obtained from fossil resources: isoprene can be prepared under mild conditions by selective catalytic conversion of prenol, which is an allylic alcohol that can be synthesised from renewable sources using metabolically engineered *Escherichia coli* (Kothandaraman et al.). Moreover, through the practice of green and sustainable chemistry, processes that use smaller amounts of energy can be conceived. Practical Mg-ion batteries require highly reversible Mg deposition and large current density, therefore both identification and removal of impurity in glyme solvents are crucial to achieve reversible Mg electrochemistry. Yang et al. were able to reach this goal, employing a facile conditioning protocol to further improve the performance of MgTFSI2/Gx electrolytes.

### Inorganic chemistry

Among the most relevant and active Inorganic Chemistry research areas, the search for biological activity of coordination compounds is a mature, yet constantly growing field. In particular, there is an urgent need for the development of new classes of compounds due to acquired resistance or limitations of the currently approved drugs against cancer.

In this Research Topic, we highlight the ability of coordination compounds such as hydrazone copper (II) complexes to behave as antiproliferative agents. Correia and co-workers managed to synthesise new copper (II) complexes bearing benzohydrazide-hydrazone derivatives of 8-hydroxy-2-quinolinecarbaldehyde and evaluate their behaviour in malignant melanoma (A-375) and lung (A-549) cancer cells. All the new compounds appeared to be potent on cancer cells, albeit with varying efficiencies in cell lines of different origins. Globally this group of complexes is more active against the melanoma cells (A-375), than in lung (A-549) cancer cells, but in both cases the IC_50_ values are in the low μM range.

### Medicinal and pharmaceutical chemistry

The primary goal of a medicinal chemist is to discover hit compounds and then hit to lead optimization with ideal selectivity towards the targets such as enzymes, proteins, receptors, DNA, and RNA which play a crucial role in disease pathways. Based on the structure and function of a suitable target, hit molecules can be designed with chosen potency, selectivity, and optimal ADME/T parameters. To date, a wide class of small molecules including natural products/their metabolites and synthetic molecules have been designed or discovered which play a crucial role in various therapeutic areas.

Natural polyphenolic small molecules such as flavonoids possess antioxidant as well as prooxidant properties. Lee et al. reported that flavonoids might affect copper transport and offer new therapeutic development. Listro et al. reviewed urea-based molecules as anticancer agents and highlighted the relevance of the urea moiety in the medicinal chemistry scenario of anticancer drugs. Ribeiro et al. synthesized Cu-complexes with hydroxyquinoline hydrazones and tested them for anticancer activities in melanoma and lung adenocarcinoma cell lines. These Cu-complexes exhibit promising anticancer activities in the low micromolar range and induce oxidative and DNA damage. Small molecules have the potential to interact with nucleic acid secondary structures such as G-quadruplexes (G4s) offering a new direction in the development of therapeutics. Besides small molecules, macrocyclic molecule, and protein-mimetic peptides (PMPs) are reported to display significant pharmacological properties that have potential in therapeutics development. Despite of Research Topic of large compounds, still there is a large chemical gap to discover new small chemical molecules with improved potency, efficacy and selectivity, and target identification. Due to the highly heterogeneous nature of various disease phenotypes and the complex mechanism of action of small molecules in biological systems, the combination approach of potential clinical candidates with existing drugs might be a suitable therapeutic application.

### Nanoscience

The aim of the Research Topic: Women in Chemistry 2022 is to highlight the significant contributions from women researchers. The study by Vlaicu et al. of the Research Topic “*Atomic scale insight into the decomposition of nanocrystalline zinc hydroxynitrate toward ZnO using Mn* ^*2+*^ *paramagnetic probes*” guides to the right selection of the most appropriate zinc hydroxynitrate synthesis method. Promising compound that exhibits a range of particular properties: anion-exchange, intercalation capacity and biocompatibility, becoming useful for a wide variety of applications in fields from nanotechnology to healthcare and agriculture. The study represents a model for the right choice of synthesis for specific applications and in the development of new green, cost-effective synthesis routes for Mn^2+^ doped nano-ZnO.

Another important study collected on this Research Topic by Cardoso et al. is represented by “*Rational design of potent ultrashort antimicrobial peptides with programmable assembly into nanostructured hydrogels*”. The always important question of the microbial resistance to common antibiotics is threatening to cause the next pandemic crisis and in this special section, the authors reported a study on the bi-functional rational design of Fmoc-peptides as both antimicrobial and hydrogelator substances for the fast, adaptable, and cost-efficient antimicrobial peptide design with programmable physicochemical properties.

The last contribution to the Nanoscience section of this Research Topic is represented by the original research by Tiwari et al., entitled “*Overcoming the rise in local deposit resistance during electrophoretic deposition via suspension replenishing*”. A promising method is represented by Electrophoretic deposition for the scalable assembly of colloidally stable nanomaterials into thick films and arrays. The study highlights an important effect, namely, that the growth of the ion-depletion region plays the most significant role in the increase of the deposit resistance, demonstrating also a method to maintain constant deposit resistance in Electrophoretic deposition by periodic replenishing of suspension, improving its scalability.

### Organic chemistry

Following the celebration of International Women’s Day 2022, the UNESCO International Day of Women and Girls in Science, *Frontiers in Chemistry* is proud to offer this platform to promote the work of women scientists, across all branches of Organic Chemistry.

At present, less than 30% of researchers worldwide are women. Long-standing biases and gender stereotypes are discouraging girls and women away from science-related fields, and STEM research in particular. Organic Chemistry is no exception to this. Science and gender equality are, however, essential to ensure sustainable development as highlighted by UNESCO. In order to change traditional mindsets, gender equality must be promoted, stereotypes defeated, and girls and women should be encouraged to pursue STEM careers.

This Research Topic has the aim of bringing into the spotlight the contributions of female researchers in all areas of Organic Chemistry. The works presented here include applications in synthesis, and modification of immobilization techniques and hydrogels and demonstrate the diversity of research performed in Organic Chemistry.

The work by Villamil et al. describes advances in thiol-thioester chemistry. The team explored the use of 2-aminothiols for the deprotection of thiols bearing an acyl group. The best results were obtained using cysteamine or L-cysteine in an aqueous buffer pH 8 at room temperature for 30 min. This process demonstrates that this deprotection can take place under very mild conditions and can yield inhibitors of a wide-range of metallo-β-Lactamases.

The work by Santis et al. describes the immobilization of an enzyme that catalyzes the regio- and stereospecific hydroxylation of non-activated C–H bonds at the only expense of H_2_O_2_. The team investigated both covalent and ionic immobilization and metal affinity binding and was found to lead to high immobilization yields and result in significantly greater stability for a peroxygenase.

The review by Wolfel et al. discusses current strategies for ligand bioconjugation to poly (acrylamide) gels with respect to chemo-selectivity and site-specificity of attachment as well as preservation of ligand’s functionality. These biomaterials have many advantages such as convenient synthesis, desirable mechanical properties within physiological range of native soft tissues, the potential to be functionalized with ligands to support the culture of living cells, and their optical transparency making them compatible with microscopy investigations. The review focuses on the application of materials for cell culture studies including specific functions of these hydrogels, and how user-friendly they are compared to the chemoselectivity the hydrogels exhibit.

### Supramolecular chemistry

Supramolecular chemistry enables the design and synthesis of multiple compounds and functional materials, leading to advancements in interdisciplinary fields such as chemistry, physics, materials science, biology, drug delivery, and nanotechnology. The papers published in this section are an example of the multidisciplinarity of the field, focusing on the dynamics and structural characterization of supramolecular complexes, peptide-based aggregates and fluorescent gels.

Novo and Al-Soufi described the evaluation of thermodynamic parameters, dynamics of supramolecular association and structural information of supramolecular complexes by fluorescence correlation spectroscopy (FCS). FCS is very useful for studying the dynamics in supramolecular host-guest complexes, when the relaxation time of the complex formation is faster than the observation time (fast-exchange dynamics), in opposition to the slow-exchange regime, which is vital for the understanding of supramolecular complexes and their function.

Fasola et al. identified a protein mimetic peptide (PMP) from *Pseudomonas aeruginosa* YeaZ protein. This PMP has the ability to switch to different secondary structural elements (α-helix, β-sheet), and to form different aggregate types (helical nanofibers, 2D-sheets and hydrogel) by changing external conditions such as concentration, hydrophobicity and temperature. PMP is a promising candidate for the generation of improved biocompatible and responsive materials from a single building block.

Impresari et al. developed different AIEgen-based luminescent hybrid supramolecular structures comprising of tetraphenylethylene scaffold and fatty acids of different chain lengths in water. The presence of longer alkyl chains facilitates the formation of stable emissive aggregates. N-terminus capping agents were used in the development of cell-viable peptides that self-assembled into luminescent fibrillary materials, which were able to form supramolecular gels in aqueous solution. Considering the widespread importance of luminescent materials, these fluorescent gels can be used as suitable substrates for *in vitro* models and bio-imaging studies.

This section further aims to encourage more women to pursue careers in this field.

### Theoretical and computational chemistry

In the continually evolving landscape of scientific research, it’s undeniable that women have made remarkable strides, particularly in the field of Chemistry. Beyond their invaluable contributions, women continue to challenge the *status quo*, driving innovation, and inspiring future generations of researchers. We recognize and salute these brilliant minds, focusing on four recent theoretical and computational chemistry contributions span across diverse Research Topic, fragment library design, spectral denoising, SARS-CoV-2 target proteins study and encapsulating population analysis.

Our journey begins with the work of Imbernon et al., who effectively harnessed the wealth of data in the Protein Data Bank to inspire fragment library design for drug discovery. The team identified fragments with multiple binding sites and modes, unravelling the potential of these multi-targeting fragments to provide frequent hits in screening, thus redefining modern medicinal chemistry.

Switching gears, Iyengar et al. took a profound leap into the fight against COVID-19. Using their expertise in computational chemistry, the team investigated alternative target sites for molecular probe design on three SARS-CoV-2 proteins, uncovering key interactions that contribute to the viral proteins’ function. These findings have the potential to revolutionize our approach to battling such an unpredictable and lethal virus.

Continuing on a biomedical front, Bian et al. and her colleagues unravel the usage of spectral denoising in improving data analysis. The team developed a novel spectral denoising method utilizing the Hilbert–Huang transform and F-test, resulting in superior results to conventional methods. This advancement is critical in modern science, with potential applications spanning from chemical investigations to medical diagnostics.

Lastly, we delve into the field of population analysis with the contribution from North et al. In their notable work, they examined the impact of basis sets and quantum methodology choices on population analysis. The results shed light on the significant variations in atomic charges based on adopted approaches, thereby highlighting the importance of careful method selection in population analysis studies.

Collectively, these works underscore the diversity of applications for computational and theoretical chemistry, from drug discovery and molecular probe design to enhanced data analysis methods.

In light of their achievements, we emphasize the growing importance and need for diversity in the scientific community. We hope that highlighting these contributions by women will inspire future generations of scientists, underscoring that gender should never be a barrier to innovation and discovery. As we move forward, let’s continue to celebrate and encourage more women in Chemistry, the echoes of their impact reverberating within the scientific community and beyond.

## Conclusion

We have included some highlights from various research activities in the field of chemistry. We anticipate that this will be a useful reference tool for students and researchers working in various areas of chemistry who are conducting chemical studies of novel systems with potential applications. This Research Topic discusses current and promising trends in chemical, biological, theoretical and computational chemistry for a sustainable and green future. The authors present contributions with original research articles, mini and full reviews, and papers on related Research Topics in this Research Topic. Electrochemical, inorganic and supermolecular chemistry are covered in the Research Topic. Researchers can refine their studies in the search by using the information provided here. Moreover, this outstanding Research Topic of manuscripts emphasises the ongoing and powerful role that nanoscience plays in our understanding of complex processes. The contributions made by the development and application of novel chemistry research tools are numerous. The work described in the manuscripts also demonstrates an acceleration in the translation of research diagnostics, and potential applications in chemistry. The reader is expected to enjoy and value the contributions of Women in Chemistry. We would like to thank all authors, reviewers, and Editorial Board members for their significant contributions to the implementation of this special Research Topic.

